# Genome-Wide Systematic Characterization of the *NPF* Family Genes and Their Transcriptional Responses to Multiple Nutrient Stresses in Allotetraploid Rapeseed

**DOI:** 10.3390/ijms21175947

**Published:** 2020-08-19

**Authors:** Hao Zhang, Shuang Li, Mengyao Shi, Sheliang Wang, Lei Shi, Fangsen Xu, Guangda Ding

**Affiliations:** Microelement Research Centre, National Key Laboratory of Crop Genetic Improvement, Key Laboratory of Arable Land Conservation (Middle and Lower Reaches of Yangtze River), Ministry of Agriculture and Rural Affairs, Huazhong Agricultural University, Wuhan 430070, China; hao.zhang@webmail.hzau.edu.cn (H.Z.); shuangli@webmail.hzau.edu.cn (S.L.); myshi@webmail.hzau.edu.cn (M.S.); sheliangwang2017@mail.hzau.edu.cn (S.W.); leish@mail.hzau.edu.cn (L.S.); fangsenxu@mail.hzau.edu.cn (F.X.)

**Keywords:** *Brassica napus*, *NPF* gene family, genome-wide, subcellular localization, expression profile, nutrient stress, transcriptional regulation

## Abstract

NITRATE TRANSPORTER 1 (NRT1)/PEPTIDE TRANSPORTER (PTR) family (NPF) proteins can transport various substrates, and play crucial roles in governing plant nitrogen (N) uptake and distribution. However, little is known about the *NPF* genes in *Brassica napus*. Here, a comprehensive genome-wide systematic characterization of the *NPF* family led to the identification of 193 *NPF* genes in the whole genome of *B. napus*. The *BnaNPF* family exhibited high levels of genetic diversity among sub-families but this was conserved within each subfamily. Whole-genome duplication and segmental duplication played a major role in *BnaNPF* evolution. The expression analysis indicated that a broad range of expression patterns for individual gene occurred in response to multiple nutrient stresses, including N, phosphorus (P) and potassium (K) deficiencies, as well as ammonium toxicity. Furthermore, 10 core *BnaNPF* genes in response to N stress were identified. These genes contained 6–13 transmembrane domains, located in plasma membrane, that respond discrepantly to N deficiency in different tissues. Robust *cis*-regulatory elements were identified within the promoter regions of the core genes. Taken together, our results suggest that *BnaNPFs* are versatile transporters that might evolve new functions in *B. napus*. Our findings benefit future research on this gene family.

## 1. Introduction

Nitrogen (N), as a key structural component of amino acid, nucleic acid, chlorophyll, and among other biologically important molecules, is a plant essential macronutrient. In plants, N can exist in several forms, including nitrate (NO_3_^−^), ammonium (NH_4_^+^), and organic molecules, mainly amino acids [1]. In aerobic soils, NO_3_^−^ is usually the most abundant source of N due to intensive nitrification processes [2]. NO_3_^−^ can be absorbed by plant roots through nitrate transporters (NRTs) and then translocated in plants [3]. An increasing number of NRTs have been identified and functionally characterized, since the first eukaryotic NRT gene was isolated almost 30 years ago from the fungus [4,5]. Generally, 4 protein families including NITRATE TRANSPORTER 1 (NRT1)/PEPTIDE TRANSPORTER (PTR) family (NPF), NRT2, CHLORIDE CHANNEL (CLC) family, and SLOWLY ACTIVATING ANION CHANNEL are involved in nitrate transport [6,7]. In recent studies, the functions of *NPF* and *NRT2* family genes in nitrate uptake and transport in different plant species have been revealed [6]. The NO_3_^−^ uptake system consists of the low-affinity transport system (LATS) and the high-affinity transport system (HATS) in higher plants [3]. Many NPFs are suggested to function as the main components of the LATS for NO_3_^−^ at high concentrations, while NRT2 family are identified as the high-affinity NRT [8]. However, some NPF proteins serve as dual-affinity transporters involved both in HATS and LATS. For example, *NPF6.3* (also named *NRT1.1* or *CHL1*), which was identified as the first plant NRT from *Arabidopsis* by screening chlorate resistance with T-DNA insertion mutants, has both low- and high-affinity nitrate uptake activities [9,10]. In comparison to their animal and bacterial counterparts, the plant NPF proteins can transport not only NO_3_^−^, but also diverse components as substrates, including dipeptides, chloride, glucosinolates, amino acids, as well as several plant hormones including auxin (IAA), abscisic acid (ABA), gibberellins (GAs), and/or jasmonates (JAs) [6,11].

Plants uniquely host huge numbers of *NPF* genes. For example, there are 53 and 93 *NPF* genes in *Arabidopsis* and rice, respectively. These genes can be divided into 8 sub-families by phylogenetic analysis [5,7]. *AtNPF4.6/NRT1.2* and *AtNPF6.3*/*NRT1.1* play major roles in NO_3_^−^ uptake at high concentrations [9,10,12]. Unlike *AtNPF6.3*, *AtNPF4.6* is a pure low-affinity NRT and is constitutively expressed [12]. *AtNPF2.7* was identified as low-affinity NO_3_^−^ excretion transporter 1 (NAXT1) located in root plasma membrane [13]. Once NO_3_^−^ is taken up by plant roots, it can be assimilated or stored in roots or transported to shoots. *AtNPF2.3* and *AtNPF7.3/NRT1.5* can facilitate root-to-shoot NO_3_^−^ transport [14,15], while *AtNPF2.9/NRT1.9* and *AtNPF7.2/NRT1.8* mediate NO_3_^−^ retrieval from xylem and phloem [16,17]. In addition to leaf NO_3_^−^ allocation, the *NPF1* members including *AtNPF1.1/NRT1.12* and *AtNPF1.2/NRT1.11*, can redistribute NO_3_^−^ into developing tissues [18]. Similarly, *AtNPF2.13/NRT1.7* is responsible for NO_3_^−^ remobilization from old leaves into young leaves [19]. *AtNPF6.2/NRT1.4*, which is highly expressed in the petiole and midrib of leaves, mediates NO_3_^−^ storage in the petioles to affect leaf development [20]. *AtNPF2.12/NRT1.6* and *AtNPF5.5* are involved in NO_3_^−^ transport into developing embryos and N storage during seed development [21,22]. Besides NO_3_^−^ transport, some NPF members, such as *AtNPF3,* were identified as GA transporters affecting GA accumulation in root endodermis [23]. In addition, recent studies have revealed the homologs of *AtNRT1.1* in rice and maize also display nitrate transport activity, indicating a conserved function of *NRT1.1* in nitrate uptake and/or transport across different species [24,25]. Interestingly, *OsNRT1.1B*, the functional homologue of *AtNRT1.1* in rice, can function not only in mediating nitrate signal transduction from the plasma membrane to the nucleus, but also in integrating the nitrate and phosphate signaling networks, as well as in regulating the root microbiota to facilitate organic N mineralization in soil [26,27]. Very recently, Wang et al. (2020) found that *OsNPF4.5* plays a key role in mycorrhizal NO_3_^−^ acquisition [28]. These studies suggest that NPFs play important roles in plant developmental processes. However, our knowledge of their specific functions is still limited, especially in plant species other than rice and *Arabidopsis*.

*Brassica napus* (genome AACC, 2n = 38), an important oil crop worldwide, is formed by recent allopolyploidy between ancestors of *B. oleracea* (Mediterranean cabbage, genome CC, 2n = 18) and *B. rapa* (Asian cabbage or turnip, genome AA, 2n = 20), which results in the genome size of *B. napus* being more than six times larger than that of *Arabidopsis* [29]. *B. napus* needs high amount of N to maintain normal growth and development, and is extremely susceptible to N deficiency. N shortage in the soil may inhibit the growth of *B. napus*, and its yield production and quality [30,31]. NPF families have been identified in various plant species, such as poplar [32], wheat [33], legumes [34], apple [35] and sugarcane [36]. However, little is known about the *NPF* family genes in oil crops such as *B. napus*. In this study, we identified the *NPF* family members in *B. napus* using the BLASTP search based on the *Arabidopsis* NPF protein sequences and investigated the characteristics and expression profiles in response to various nutrient supplies. Moreover, coexpression networks and subcellular localization were analyzed to reveal expression pattern and molecular mechanism across the *NPF* genes in *B. napus*. Our results present a comprehensive characterization of the *BnaNPF* family genes, and offer the foundation in the complex genetic dissection of the NO_3_^−^ transport system in *B. napus*.

## 2. Results

### 2.1. Genome-Wide Identification of the NPF Family Genes

To identify the *NPF* family members in *Brassica* species, we used the amino acid (aa) sequences of AtNPFs to perform BLASTP search against the genome databases of *B. rapa* (“Chiifu-401”), *B. oleracea* (“TO1000”), and *B. napus* (“Darmor-*bzh*”) according to the homology with 53 NPF proteins in *Arabidopsis*. Finally, a total of 95, 93 and 193 *NPF* family genes were identified in *B. rapa*, *B. oleracea* and *B. napus*, respectively (Table 1). Large differences in the *NPF* homolog number occurred during the evolutionary process of *Brassica* species. The number of *NPF* genes in *B. rapa*, *B. oleracea* and *B. napus* was 1.8, 1.8 and 3.6 times that in *Arabidopsis*, respectively. Furthermore, the number of N*PFs* in *B. napus* was similar to the sum of *NPFs* both in *B. rapa* and *B. oleracea*, suggesting that most of the *NPFs* were retained during the alloploidy formation of *B. napus*. However, some of the homologues disappeared or were additionally duplicated in *B. napus* after hybridization between *B. rapa* and *B. oleracea*, as the number of *NPFs* in some chromosomes of *B. napus* A and C subgenomes differed from that in *B. rapa* and *B. oleracea*. In *B. napus*, the number of each *NPF* subfamily varied from 6 to 73 with an average of more than 24 homologs. *BnaNPF5* subfamily has the largest number of members, followed by *BnaNPF2*, *BnaNPF8*, *BnaNPF4*, correspondingly. On the contrary, *BnaNPF3* subfamily has the smallest number. The differentiation in the number of the *NPF* subfamily might suggest differential expansion patterns of *NPFs* during the allopolyploidy process of *B. napus*.

### 2.2. Phylogenetic Analysis, Gene Structure and Conserved Motif Analysis of BnaNPFs

**Figure 1:**
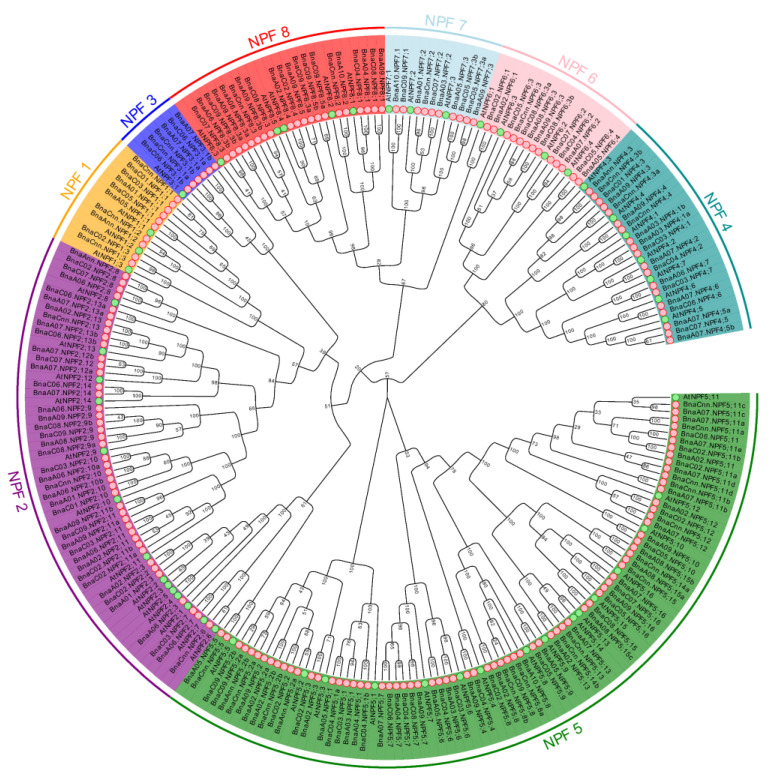
Phylogenetic tree of coding nucleotide sequences of the NITRATE TRANSPORTER 1 (NRT1)/PEPTIDE TRANSPORTER (PTR) family (NPF) in *Brassica napus* and *Arabidopsis thaliana*. The phylogenetic tree was constructed by Molecular Evolutionary Genetics Analysis (MEGA) 5.1 with neighbor-joining method and 1000 replicates. A total of 246 nucleotide sequences including 193 from *B. napus* (pink circular), and 53 from *Arabidopsis* (green circular) were involved in the analysis.

To elucidate the molecular evolution and phylogenetic relationships among the NPF proteins, we constructed an unrooted phylogenetic tree including 53 AtNPFs and their homologs in *B. napus* (Figure 1). The results showed that the NPF homologs could be divided into 8 clades, and the gene duplication events of *NPF* in *B. napus* were either after or parallel to *Arabidopsis* formation. To distinguish the subfamilies of *NPF* genes, we renamed all the *BnaNPF* homologues according to the homology with *AtNPF* genes following the international nomenclature for *B. napus* genes (Figure 1, Table S2). We further analyzed the gene structure of *NPFs* in *B. napus* and *Arabidopsis* (Figure S1). The results showed that most of the *NPF* genes in *B. napus* contained 2 to 5 introns, especially *NPF7* subfamily, and most genes (45.6%) had 3 introns. It is noteworthy that the *BnaNPF* family members had similar gene structure with their counterparts in *Arabidopsis*, especially *NPF2;13*, *NPF4;2*, *NPF5;3*, *NPF5;10*, *NPF8;2* subgroups. In addition, we obtained conservative motifs of the *NPF* family genes in *Arabidopsis* and *B. napus* (Figure S2). Nine motifs were identified from 246 *NPF* genes in *Arabidopsis* and *B. napus*. Nearly all the *NPF* genes contained Motif 1, Motif 3, Motif 5, Motif 6, Motif 7 and Motif 9. Conversely, very few genes contained Motif 2 and Motif 8, especially Motif 8 which mainly appeared in *NPF5* subfamily. Moreover, most of the closely related genes in each subgroup shared similar motif composition, but it varied largely among different subfamilies, except *NPF2;13*, *NPF4;2*, *NPF5;3*, *NPF5;10*, *NPF8;2* subgroups. The similar motif arrangements among NPF proteins within subgroups indicated that the protein architecture of NPF was very conservative within a specific subfamily.

### 2.3. Molecular Characterization of BnaNPFs

**Figure 2:**
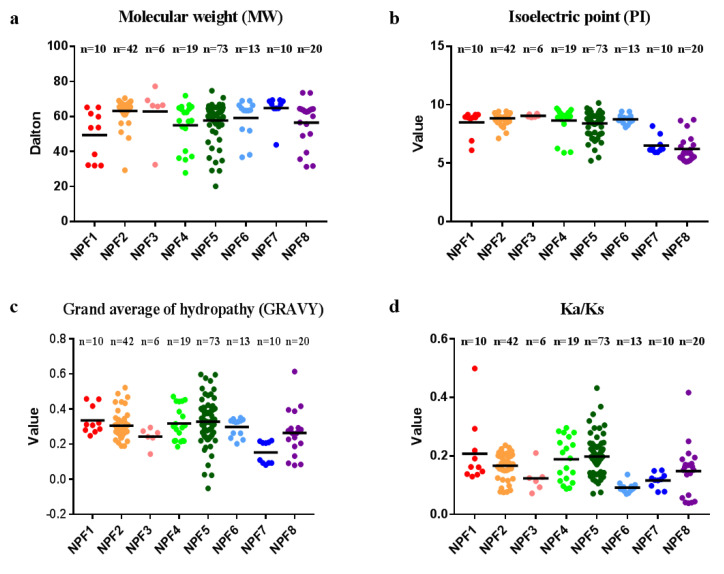
Molecular characterization of the NPF proteins in *Brassica napus*. (**a**) molecular weight (MW); (**b**) theoretical isoelectric point (PI); (**c**) grand average of hydropathy (GRAVY); (**d**) Ka/Ks values.

To unravel the molecular characteristics of the BnaNPF proteins, we calculated molecular weight (MW), isoelectronic points (PIs), and grand average of hydropathy (GRAVY) of each BnaNPF protein using the ProtParam tools (Figure 2, Table S2). The results revealed that the genomic DNA length of the BnaNPF without the untranslated region varied from 549 bp to 2744 bp, while the protein length ranged from 182 aa to 692 aa. MW, which is related to protein length, ranged from 20.14 kDa to 77.23 kDa (Figure 2a, Table S2). Most proteins in the same subfamily had similar parameters with some exceptions, such as *BnaA07.NPF4;5b* and *BnaA07.NPF4;6*, but proteins in different subfamilies were greatly discrepant with each other. The theoretical PIs of BnaNPFs varied from 5.12 to 10.16. Moreover, about 80% of the NPF family members in *B. napus* have high isoelectric points (PI > 7), including most of the proteins in NPF1 to NPF6 subfamilies (Figure 2b, Table S2). The GRAVY value of BnaNPF proteins, which is calculated as the sum of aa hydropathy values divided by the protein length, varied from −0.051 to 0.615, indicating that all the BnaNPF family proteins are hydrophobic (GRAVY > 0), except *BnaC02.NPF5;2* (Figure 2c, Table S2). The prediction of the subcellular localization using online WoLF PSORT indicated that most of the *BnaNPFs* were localized on the cell membrane, suggesting that they might be responsible for the trans-membrane transport of certain substates (Table S2).

In addition, to characterize selection pressure on the *BnaNPFs* during the evolutionary process, we used the orthologous *NPF* gene pairs between *Arabidopsis* and *B. napus* to determine the values of synonymous (Ks) and nonsynonymous (Ka) nucleotide substitution rates, and Ka/Ks ratios (Figure 2d, Table S3). In general, a Ka/Ks ratio greater than one means positive selection, whereas a ratio less than one indicates a functional constraint, and a ratio equal to one means neutral selection [37]. Our results showed that all the Ka/Ks ratios of the orthologous *NPF* family genes between *Arabidopsis* and *B. napus* were <1, indicating that the *BnaNPFs* might suffer from strong purifying selection for retention.

### 2.4. Chromosomal Location and Duplication Pattern Analysis of BnaNPFs

Chromosomal location analysis showed that 193 *BnaNPF* genes scattered across 19 chromosomes, including 91 genes located on the A subgenome and 102 genes located on the C subgenome (Figure S3). Five and 29 genes, which were located on the A and C subgenomes respectively, could not be mapped to a specific chromosome. Some chromosomes had relatively many genes, whereas others had relatively few. In the A subgenome, chromosome A07 has the highest number (21) of the *NPF* genes, followed by A09 (16 genes), while chromosomes A04 and A10 had the least members (3 genes). In the C subgenome, chromosome C02 has the most members (13 genes) of the *BnaNPFs*, while chromosome C01 has only 3 genes. 

**Figure 3:**
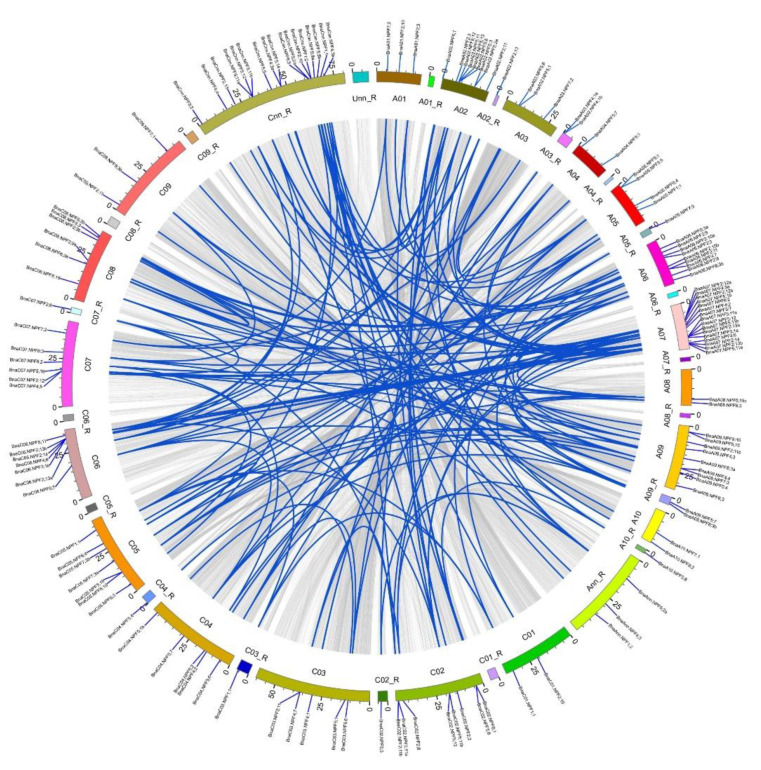
Schematic representations for the chromosomal distribution and interchromosomal relationships of rapeseed *NPF* genes. Gray lines in the background indicate all syntenic blocks in the *Brassica napus* genome, and the blue lines indicate syntenic NPF gene pairs. The chromosome number is indicated at the bottom of each chromosome. R, random.

Gene family expands mainly via three pathways: tandem duplication, segmental duplication, and whole-genome duplication [38]. In order to further uncover the evolution processes of the *BnaNPF* family, we analyzed the gene replication patterns using their CDS sequences (Figure 3). A chromosomal region within 200 kb containing two or more homologous genes is defined as a tandem duplication event [39]. In this study, we found 169 segmental duplication events including 137 *BnaNPF* family genes using MCScanX. Only *BnaA03.NPF4;1a* and *BnaA03.NPF4;1b* were identified as a tandem duplication event, which was 6.5 kb apart (Figure 3). There were more segmental duplication events on chromosomes A02 and A07 compared to other chromosomes, including 20 and 41 genes, respectively. Therefore, compared with tandem replication, segmental duplication might be the main driving force for the amplification of the *BnaNPF* family.

**Figure 4:**
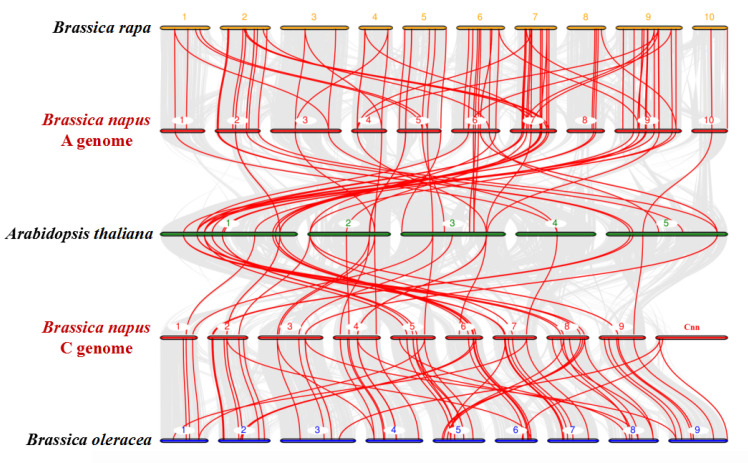
Synteny analysis of the *NPF* genes in *Brassica napus*, *B. rapa*, *B. oleracea* and *Arabidopsis thaliana* chromosomes. Gray lines in the background indicate the collinear blocks within *B. napus* and other plant genomes, while the red lines highlight the syntenic NPF gene pairs. Genes located on *B. napus* A genome are syntenic with genes of *B. rapa* and *A. thaliana*, while genes located on *B. napus* C genome are syntenic with genes of *B. oleracea* and *A. thaliana*.

In the current research, we identified 95 and 93 *NPF* genes in the *B*. *rapa* and *B*. *oleracea* genomes (Table 1), respectively. To further infer the phylogenetic mechanisms of the *B*. *napus NPF* family, we constructed a comparative syntenic map of *B*. *napus* and its ancestors (*Arabidopsis*, *B*. *rapa* and *B*. *oleracea*). The results revealed that there were strong orthologs of *NPF* genes between *B. napus* and the other three ancestral species (Figure 4). Among them, there were 52 and 84 pairs of syntenic relationships in the A subgenome of *B. napus* with *Arabidopsis* and *B. rapa*, respectively. In the C subgenome of *B. napus*, there were 41 and 70 pairs of syntenic relationships with *Arabidopsis* and *B. oleracea*, respectively. Interestingly, most syntenic relationships of the *NPF* genes between *B. napus* and *Arabidopsis* are mainly located on *Arabidopsis* chromosome 1, and these genes were interconnected with *BraNPFs* and *BolNPFs*. These results suggested that whole-genome duplication (polyploidy) was another important driving force for *NPF* gene evolution in *B. napus*.

### 2.5. Transcriptional Profiles of BnaNPFs under Diverse Nutrient Stresses

**Figure 5:**
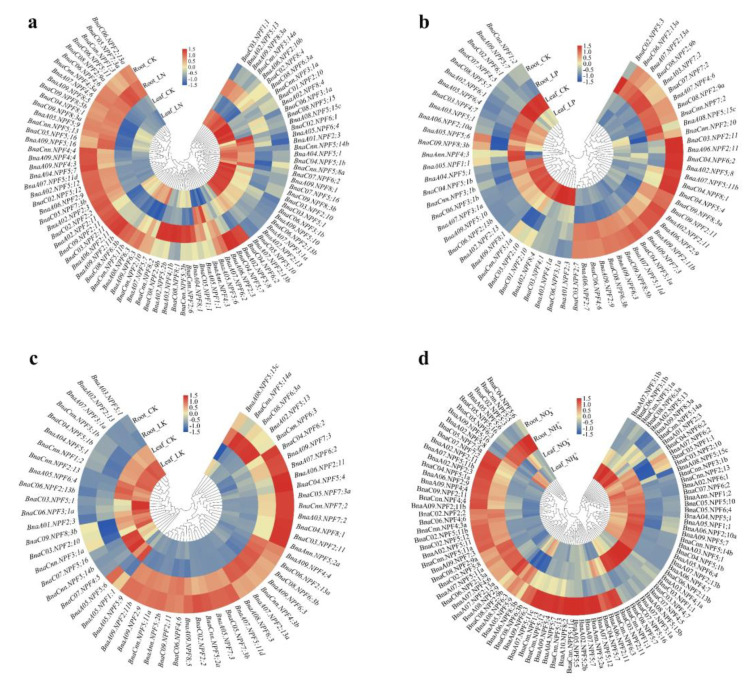
Expression profiles of the NPF family differentially expressed genes (DEGs) in the leaves and roots of *Brassica napus* under nitrogen (N), phosphorus (P), potassium (K) stress (**a**–**c**) and ammonium toxicity (**d**) environments. For nutrient stress treatments, 14-day-old seedlings were exposed to N, P and K for seven days. For ammonium toxicity assay, 14-day-old seedlings were treated with 6 mM NO_3_^−^ (control) and NH_4_^+^ only for nine days. The fully expanded leaves and roots were sampled separately for RNA-seq analysis. CK, normal nutrient supply. LN, N deficiency. LP, P deficiency. LK, K deficiency. NO_3_^−^, NaNO_3_. NH_4_^+^, NH_4_Cl. The color scale is shown in the middle. Heat maps of gene expression profiles were generated using TBtools after data normalization (Z-score).

To identify the roles of *BnaNPFs* in regulating rapeseed against various nutrient supplies, we investigated their transcriptional responses under N, phosphorus (P), potassium (K) deficiencies, and ammonium toxicity environment based on RNA-seq data (Table S4). The results showed that the expression profiles of *BnaNPF* genes varied largely in response to different nutrient levels (Figure 5, Table S4), indicating their complex roles in the growth regulation of *B. napus* under diverse nutrient conditions. Under N starvation, the expression profiles of the *BnaNPF* genes were significantly altered (Figure 5a). Compared to the N sufficient condition, 65 and 47 *BnaNPFs* in leaves and roots were differentially expressed, respectively. Approximately 84.6% (55/65) differentially expressed genes (DEGs) were significantly upregulated in leaves under N limitation condition, while only 44.7% (21/47) DEGs in roots were significantly upregulated. Among them, 11 *BnaNPF* genes were significantly induced and only two genes (*BnaC02.NPF2;2* and *BnaC04.NPF6;2*) were inhibited both in leaves and roots. Under P starvation, 42 and 31 *BnaNPF* DEGs in leaves and roots were identified, respectively (Figure 5b). In leaves, 28 *BnaNPF* genes were significantly upregulated by P stress, and 14 *BnaNPF* genes were significantly downregulated. In roots, 21 and 10 *BnaNPF* DEGs were induced and repressed under P deprivation, respectively. Four *BnaNPF* genes were significantly upregulated and two genes (*BnaA05.NPF1;1* and *BnaC09.NPF8;3b*) were downregulated both in leaves and roots. Under K stress, the expression of 22 and 43 *BnaNPF* genes were significantly altered in leaves and roots, respectively (Figure 5c). Moreover, the expression of four *BnaNPF* DEGs were significantly induced and two genes (*BnaA05.NPF1;1* and *BnaA09.NPF7;3*) were significantly inhibited both in leaves and roots under K stress. In addition, 51 and 72 *BnaNPF* DEGs were identified in leaves and roots respectively under NH_4_^+^ toxicity (6 mM N), which were more than other nutrient stresses (Figure 5d). Most of the *BnaNPF* DEGs were downregulated when ammonium was supplied as the sole N source, including 35 genes in leaves and 48 genes in roots. Only six genes were upregulated and 14 genes were downregulated both in leaves and roots under NH_4_^+^ supply only.

**Figure 6:**
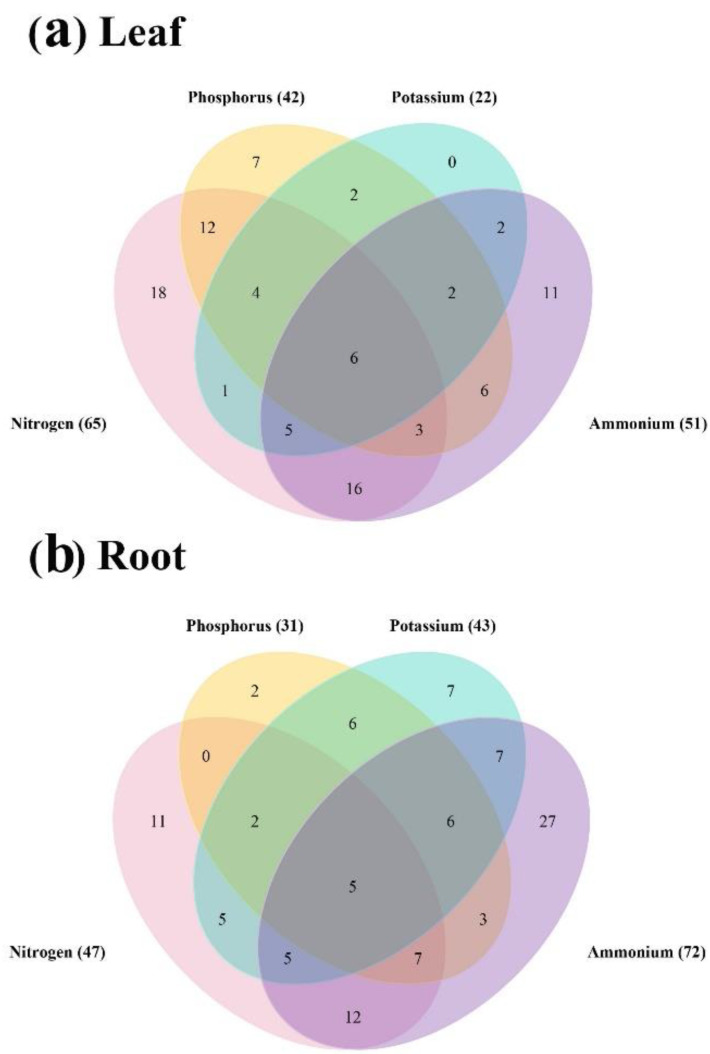
Venn diagram showing the transcriptional responses of the *BnaNPF* genes in leaves (**a**) and roots (**b**) of *Brassica napus* under diverse nutrient supplies. Each color represents a different treatment and the number in brackets represent the differentially expressed genes between the control and treatments.

To investigate whether *BnaNPF* genes can respond to diverse nutrient conditions simultaneously, a Venn diagram was constructed using the identified DEGs by TBtools (Figure 6). In leaves, 18 and seven DEGs were specially regulated by N and P stresses, respectively. Eleven DEGs were affected by sole ammonium supply. In roots, 11 DEGs were regulated by N deficiency specially, while two by P deficiency and seven by K deficiency. Twenty-seven DEGs were specially affected by ammonium-N. Moreover, the expression of six and five genes were changed simultaneously across all the environments in leaves and roots, respectively. Interestingly, one gene (*BnaA05.NPF1;1*) was identified as a DEG across all the conditions both in leaves and roots, indicating that it might play a key role in coordinating rapeseed response to multiple nutrient conditions simultaneously.

### 2.6. Identification of the BnaNPF Hub Genes and Expression Analysis Among Different Tissues

**Figure 7:**
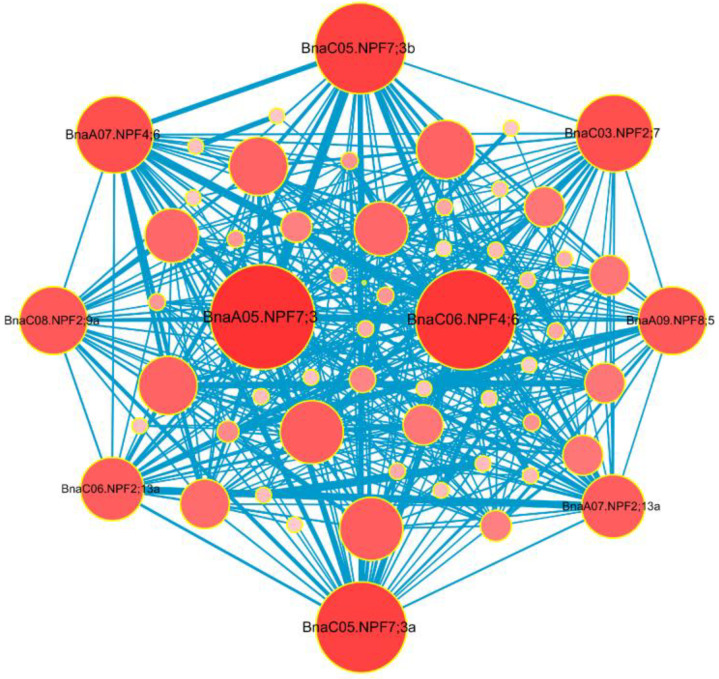
Coexpression networks of the *BnaNPF* family genes. Cycle nodes represent genes, and the size of the nodes represents the power of the interrelation among the nodes by degree value. The width of the lines between two nodes represents the strength of the interactions between two genes. The hub *NPF* genes located in the center of the network, while the 10 most coexpressed genes were displayed in network.

To identify the core *BnaNPF* genes in response to N stress, we analyzed the coexpression relationships among the *BnaNPF* genes based on Fragments per Kilobase Million (FPKM) values from RNA-seq data (Figure 7). A total of 402 pairs of coexpression relationships among 65 genes were identified from the *BnaNPF* family. The top 10 *BnaNPF* genes which have the strongest coexpression relationships with other genes were defined as the hub genes. Among them, *BnaA05.NPF7;3* interacted with 33 *NPF* genes, while *BnaC06.NPF4;6* interacted with 32 *NPF* genes. Both *BnaC05.NPF7;3a* and *BnaC05.NPF7;3b* coexpressed with 30 *NPF* genes. With respect to *BnaC03.NPF2;7* and *BnaA07.NPF4;6*, *BnaC08.NPF2;9a* and *BnaA09.NPF8;5*, *BnaA07.NPF2;13a* and *BnaC06.NPF2;13a*, each pair of these genes had high interaction intensity with 27, 25 and 24 *NPF* genes, respectively. Interestingly, *BnaA05.NPF7;3* and *BnaC05.NPF7;3b*, which have high expression level in root, were also in core position in the *BnaNPF* family. Therefore, *BnaA05.NPF7;3* and *BnaC05.NPF7;3b* might be of great importance in response to N stress in *B. napus*.

**Figure 8:**
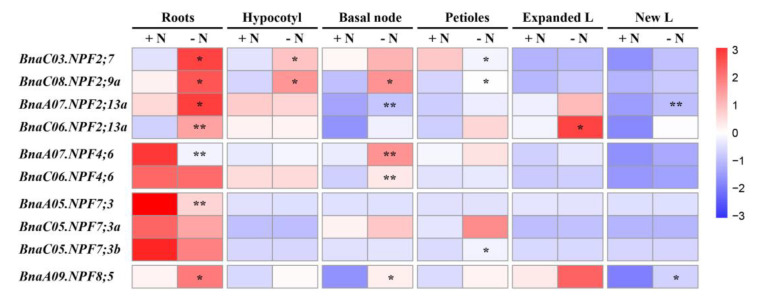
The expression profiles of the 10 core *BnaNPF* genes in different tissues under nitrogen (N) stress by quantitative Real-Time PCR (qRT-PCR). Seedlings of 14 days’ old were exposed to N-free nutrient solution for six days. The roots, hypocotyl, basal node, petioles, fully expanded leaves (expanded L) and new leaves (new L) were sampled separately for RNA extraction. CK, normal nutrient supply; LN, N stress (0 μM N) condition. The color scale was shown on the right side. The heat map was generated using TBtools after data normalization (Z-score). * and ** indicates significant difference at *P* < 0.05 and *P* < 0.01 by student’s *t* test, respectively.

To further understand the expression patterns of these core genes in response to N stress, roots, hypocotyl, basal node, petioles, fully expanded leaves and new leaves were used to investigate gene expression pattern using quantitative Real-Time PCR (qRT-PCR). The results showed that most of the core *BnaNPF* genes expressed preferentially in roots (Figure 8). The expression levels of *BnaA07.NPF4;6*, *BnaC06.NPF4;6*, *BnaA05.NPF7;3*, *BnaC05.NPF7;3a* and *BnaC05.NPF7;3b* were significantly inhibited or unchanged in roots under N stress. However, *BnaC03.NPF2;7*, *BnaC08.NPF2;9a*, *BnaA07.NPF2;13a*, *BnaC06.NPF2;13a* and *BnaA09.NPF8;5* were significantly upregulated by N deficiency in roots. These results are consistent with the changes in RNA-seq data (Table S4). Moreover, the core *NPF* genes were differentially expressed in other tissues except roots under N limitation. For example, the expression levels of *BnaC08.NPF2;9a*, *BnaA07.NPF2;13a*, *BnaA07.NPF4;6*, *BnaC06.NPF4;6* and *BnaA09.NPF8;5* were significantly induced by N deficiency in basal node, while *BnaC03.NPF2;7* and *BnaC08.NPF2;9a* were significantly upregulated by N stress in hypocotyl and petioles, and *BnaC06.NPF2;13a* were induced by N stress in fully expanded leaves.

### 2.7. Subcellular Localization and Transmembrane Domain Analysis of the BnaNPF Hub Genes

**Figure 9:**
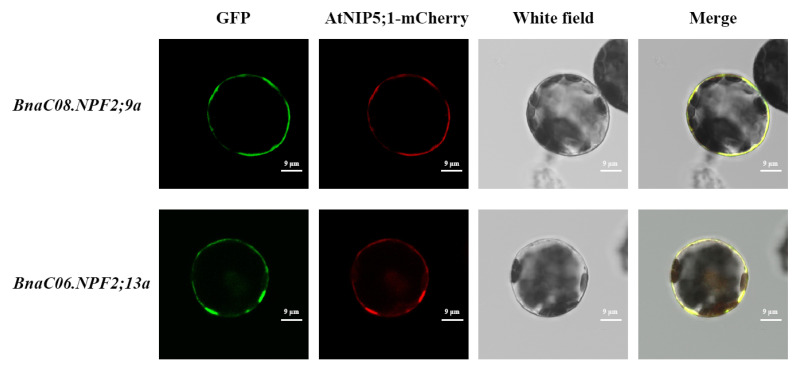
Subcellular localization of BnaC08.NPF2;9a and BnaC06.NPF2;13a. *35S*::*BnaNPFs*-*GFP* and *35S*::*AtNIP5;1*-*mCherry* constructs were introduced into *Arabidopsis* protoplasts. The GFP and mChery fluorescence was observed with a confocal laser-scanning microscope. The images were taken in the dark and white field.

Nitrate uptake is carried out by plasma membrane-located nitrate transporters, such as *OsNPF4;5* [28]. In *B. napus*, we performed the subcellular localization analyses of BnaNPF proteins using the WoLF PSORT program. The result showed that most of the BnaNPF proteins, including the core *BnaNPF* genes, were predicted to be plasma membrane localized (Table S2). To investigate the physiological roles of the BnaNPF proteins in nitrate uptake, BnaC08.NPF2;9a and BnaC06.NPF2;13a were further randomly selected to be experimentally examined for their subcellular localization in the plant cell according to the expression abundance and patterns in roots. When transiently expressed in *Arabidopsis* protoplasts, both BnaC08.NPF2;9a-GFP and BnaC06.NPF2;13a-GFP fusion protein signals were completely merged with the AtNIP5;1-mCherry fusion protein signals. This result demonstrates that the two BnaNPF proteins localize in the plasma membrane (Figure 9). Moreover, we predicted the transmembrane domains (TMDs) of the 10 core *BnaNPF* genes using TMHMM program (Figure S4). Almost all the core genes have 9–13 TMDs, which are similar with their *Arabidopsis* counterparts [8]. However, BnaC03.NPF2;7 was predicted to have only six TMDs. Thus, there might be differentiation and variation in the evolution of NPF family, which may further lead to functional differences.

### 2.8. Cis-Regulatory Element Analysis of the BnaNPF Family

**Figure 10:**
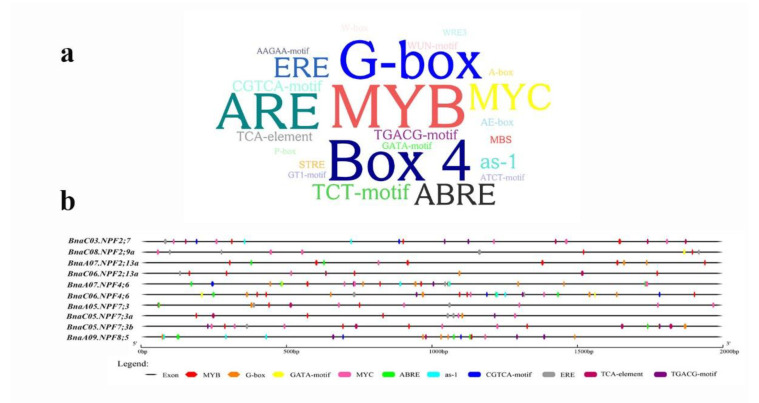
Identification of the *cis*-regulatory elements (CREs) in the 2.0-kb promoter region of the 10 core *BnaNPF* genes. (**a**) Over-presentation of the CREs in the promoters of the 10 core *BnaNPF* genes. The more the CREs, the bigger the typeface size. (**b**) Nitrogen responsive CREs (double sided wedge) and phytohormone responsive CREs (round-corner rectangle) were showed in the promoter regions of the 10 core *BnaNPF* genes. Different CREs are indicated with different colors.

Transcription factors (TFs) play important roles in transcriptional regulation of downstream genes by binding to *cis*-regulatory elements (CREs) in the promoters. To identify the core TFs regulating *BnaNPFs*, the 2.0-kb upstream sequences of the core *BnaNPF* start codons were used to identify the over-accumulated CREs. The results showed that CREs are abundant in the 2.0-kb promoter regions of the 10 core *BnaNPF* genes (Figure 10). Apart from the abundance of the common CREs including the TATA box and the CAAT box (data not shown), the MYB and G-box elements were most highly enriched in the *BnaNPF* promoter regions. These CREs have been reported to be involved in the molecular regulation of N status in plants [40,41,42]. Meanwhile, we also found a large number of phytohormone responsive CREs, including ABRE, as-1, CGTCA-motif, ERE, TCA-element, TGACG-motif and MYC elements (Figure 10b). Notably, the GATA-box was only identified in the promoters of *BnaC08.NPF2;9a*, *BnaC06.NPF2;13a*, *BnaA07.NPF4;6* and *BnaC06.NPF4;6*. These results suggested that complex regulatory networks might be involved in the transcriptional regulation of the core *BnaNPF* genes.

## 3. Discussion

### 3.1. Complicated Phylogeny of the NPF Genes in Brassica Napus

The *NPF* genes encode numerous proteins that comprise a large family of members broadly distributed in eukaryotes [32,35,43]. They facilitate transport of a wide variety of nitrogenous compounds [11,44]. *NPF* genes have been identified within many species over the last decades, such as *Arabidopsis* [8], poplar [32], *Medicago truncatula* [34], rice [5,43], apple [35] and sugarcane [36]. However, the number of *NPF* family members varies largely among species. In this research, we identified 193 *NPF* genes in the whole genome of *B. napus* genome, as well as 95 members in *B. rapa* and 93 in *B. oleracea* (Table 1 and Table S2). The number of *NPF* genes in *B. napus* is higher than that in any other species reported so far. This may be due to the reason that *Brassica* species experienced an extra whole-genome triplication event that contributed to a gene-level evolution and drove the diversification of the *Brassica* plants compared with *Arabidopsis* [29,45]. *B. napus* was derived from the recent hybridization between *B*. *rapa* and *B*. *oleracea*. One *Arabidopsis* gene should theoretically correspond to six orthologs in *B*. *napus*, and three orthologs in *B*. *rapa* and *B*. *oleracea*. However, we found that the expansion of *NPF* genes in *B. napus*, *B. rapa* and *B. oleracea* resulted in approximately 3.6, 1.8 and 1.8 times to that in *Arabidopsis*, respectively (Table 1). These results indicate that duplicated genes might have been lost during evolution, as the synteny between the *NPF* genes in *Arabidopsis* and their homologs in *B*. *napus*, *B*. *rapa* and *B*. *oleracea* was less than expected (Table 1, Figure 4). Most likely, the essential *NPF* genes were retained in *B. napus* genome during the long-term natural selection process, while the others were lost. That is to say, the whole-genome duplication raises the rate of gene gains and losses [45,46]. Furthermore, the *B. napus* genes number is slightly more than the total number of *B. rapa* and *B. oleracea* (Table 1), indicating that gene duplication and chromosome rearrangement in *B. rapa* and *B. oleracea* is expected to result in a conserved gene distribution of *NPF* in *B. napus*. This is further confirmed by the construction of the comparative syntenic map of three *Brassica* species (Figure 4). These findings implied that the whole-genome duplication play an essential role in *B. napus*, which were consistent with the *Brassicaceae* evolutionary history [47].

Tandem duplication and segmental duplication are considered to be two main pathways of gene family expansion in plants [38]. Here, we found that 137 of 193 (71%) *BnaNPFs* in the *B. napus* genome were identified as being involved in segmental duplication event, while only *BnaA03.NPF4;1a* and *BnaA03.NPF4;1b* as tandem duplication pairs (Figure 3). Segmental duplication events may widely disperse gene copies throughout the genome where they experience few recombinational exchanges with parental copies [48]. The *NPF* genes might have undergone functional divergence during evolution, as indicated by the expression profile analysis (Figure 5, Table S4). The functional differentiation of *NPF* genes in *B. napus* need to be addressed in future. In a word, *NPF* family might have complex phylogeny in the *B. napus*, which is due to the highly diversified *NPF* family and allopolyploid characteristics of the *B. napus* genome. Segmental duplication and whole-genome duplication are the main force for the expansion of the *NPF* gene family in *B. napus*, which provides a valuable basis to further evaluate gene function in nitrate uptake and translocation.

### 3.2. Conserved and Complex Structural Characteristics of NPF Genes in Brassica Napus

The gene structures of *BnaNPF*s are highly conserved within the same sub-family (Figures S1 and S2, Table S2). Similar results have been reported in *Arabidopsis* [8], rice [5], *Medicago truncatula* [34], sugarcane [36], and poplar [32]. For example, in line with the counterparts in *Arabidopsis*, most of the *NPF* genes in *B. napus* contained three introns and four exons such as *NPF2;13*, *NPF4;2*, *NPF5;3*, *NPF5;10*, and *NPF8;2* subgroup members. However, the gene structure differed in different subfamilies (Figures S1 and S2, Table S2). In terms of protein motif, most of the *BnaNPF* genes possess six conserved motifs (Figure S2), while *NPF* genes from rice and apple contain three conserved motifs [5,35]. This might suggest the functional diversity of *BnaNPF* members due to the variation in aa residues except the conserved domain. The average length of NPF proteins is 529 amino acids, while each *NPF* genes usually encoded 400 to 700 amino acids in *B. napus* (Table S2), making them similar in size to the NPF proteins in other species [8,32,35]. The NPF transporters in higher plants usually contain 12 putative transmembrane spanning regions. For example, it is reported that the *NPF* genes in *Arabidopsis* and apple possess 12 TMDs [8,35]. However, there are some NPF members in sugarcane and poplar which have TMDs less than 12 [32,36]. Here, we found that, except for *BnaC03.NPF2;7* (6 TMDs), the nine core BnaNPF proteins are predicted to contain 9 to 13 TMDs. It is similar to their *Arabidopsis* counterparts, but still marginally smaller than *Arabidopsis*. This is likely due to a relaxed selection strength as well as the limited quality for some portion of assembled genomic sequences, because many potential TMDs did not make the cutoff value to be assigned as TMDs by the software [36]. Thus, the core NPF genes in *B. napus* might have the capacity to transport nitrate, which need be uncovered using yeast mutant and transgenic approaches in future. Generally, the BnaNPF family in *B. napus* has complex and conserved structural characteristics as more members than any other species reported thus far.

### 3.3. NPFs Are Versatile Transporters That Might Evolve New Functions in Brassica Napus

NPFs belong to the major facilitator superfamily (MFS) superfamily that comprises facilitators, symporters, and antiporters [49]. Several NPF proteins are required for absorption of nitrate from external environment and transportation among cells, tissues and organs [6,7,28]. Based on the RNA-seq, we analyzed the expression patterns of the 193 *BnaNPF* genes under N stress, and 65 and 47 DEGs were identified in leaves and roots, respectively (Figure 5a), indicative of the important roles in nitrate uptake. *AtNPF6.3*/*AtNRT1.1*, the first identified nitrate transporter in *NPF* family in *Arabidopsis*, is currently the most extensively studied member in this family [6,9]. *AtNPF6.3* has both low- and high- nitrate uptake affinities, and is expressed predominantly in roots [8]. Among homologous genes of *AtNPF6;3* in *B. napus*, *BnaA09.NPF6;3* and *BnaC08.NPF6;3b* have the highest abundance under N sufficient and was significantly regulated by N stress in roots (Figure 1 and Figure 5a, Table S4), suggesting their vital roles in N uptake, as suggested by their counterparts in *Arabidopsis* [8]. Interestingly, the expression of *BnaA09.NPF6;3* and *BnaC08.NPF6;3b* were also upregulated by N deprivation in leaves, indicating that they might also function in nitrate translocation and utilization.

The orthologous *NPF* genes in other species might have evolved additional functions in comparison to *Arabidopsis* single *NPF* gene locus [50]. Compared with model plants such as *Arabidopsis*, the *NPF* family appears to have more complex functions in N uptake and transport in *B. napus*. Thus, we identified 10 core genes of *BnaNPF* family using coexpression network analysis and explored their expression patterns in response to N deprivation in more details (Figure 7 and Figure 8). Nine core NPF genes are homologous with the *Arabidopsis* genes with known functions (Figure 8). *AtNPF2.7*, located in the root plasma membrane, is involved in root NO_3_^−^ efflux to the outer medium [13], while *AtNPF2.13* is expressed in phloem of the minor veins of old leaves, which remobilizes NO_3_^−^ from old leaves into young leaves under N starvation [19]. The constitutively expressed *AtNPF4.6* is involved in low-affinity root NO_3_^−^ uptake [12]. Moreover, *AtNPF2.9* and *AtNPF7.3* are involved in root-to-shoot NO_3_^−^ translocation, affecting phloem and xylem loading, respectively [15,16]. In line with *Arabidopsis*, *BnaC03.NPF2;7*, *BnaC08.NPF2;9a*, *BnaA07.NPF4;6*, *BnaC06.NPF4;6*, *BnaA05.NPF7;3*, *BnaC05.NPF7;3a* and *BnaC05.NPF7;3b* were expressed preferentially in roots (Figure 8). The changes in the expression levels of the core *BnaNPF* genes may lead to the accumulation of NO_3_^−^ in roots, which can contribute to plant tolerance to stress conditions [51]. Subcellular localization analysis indicated that most of these genes are located in plasma membrane (Figure 8, Figure 9 and Figure S4, Table S2). In addition, *BnaC06.NPF2;13a* has a relatively high expression level in fully expanded leaves and is up-regulated by N deficiency, suggesting its similar NO_3_^−^ redistribution function to *AtNPF2.13* [19]. CRE analysis in the promoter region of the core *NPF* genes lead to the identification of some TF binding elements and phytohormone responsive elements (Figure 10), indicative of the complex regulatory networks by TF and/or phytohormones [23,52].

It is well documented that the expression of ion transporters might be involved in a process of affecting nutrient homeostasis because of the cross-talk among ion signals in response to different nutrient stresses [53]. *NPF* family is one of the most important NRTs in plants, but it can also be regulated by other nutrients, and be involved in different signal pathways [26,52,54]. For example, phosphate availability can modulate the expression of nitrate-responsive genes, and OsNRT1.1B can interact with a phosphate signaling repressor SPX4 to implement the coordinated utilization of N and P [26]. Here, we found that there were 6 and 5 genes responding to N, P and K stresses and NH_4_^+^ toxicity simultaneously in leaves and roots of *B. napus*, respectively (Figure 6). These results suggest that in addition to functioning in nitrate uptake and translocation, *BnaNPF*s might be involved in crosstalk for sensing external status of N, P, and K in *B*. *napus*. Yet, the underlying mechanisms need to be further elucidated. Previous reports show that *AtNRT1.1*/*NPF6.3* is the main factor in regulating plant response to NH_4_^+^ toxicity by affect NH_4_^+^ uptake and metabolism [55]. Here, we observed 51 and 72 *BnaNPF* DEGs in leaves and roots under NH_4_^+^ toxicity condition, including six homologous genes of *AtNRT1.1/NPF6.3*. It is noteworthy that *BnaA05.NPF1;1* identified as a DEG across all the nutrient conditions both in leaves and roots, suggesting its key role in coordinating the resistance of *B. napus* to N, P and K stresses, as well as NH_4_^+^ toxicity.

## 4. Materials and Methods 

### 4.1. Identification of the NPF Family Genes

The *NPF* family genes were identified in *B. napus* based on their homology with the 53 NPF proteins from *Arabidopsis* database in TAIR (https://www.arabidopsis.org/) using the BLASTP search program in the CNS-Genoscope genomic database (http://www.genoscope.cns.fr/brassicanapus/) [29]. Redundant sequences were removed manually. All the *BnaNPF* genes were analyzed using the Hidden Markov Model (HMM) in the Pfam database (http://pfam.sanger.ac.uk/search) to confirm that all the genes belonged to the *NPF* family. The *NPF* genes in *B. rapa* and *B. oleracea* were acquired as well using the same method described above. In addition, the genomic DNA, cDNA, CDS and protein sequences of the *NPFs* were derived from the *B. napus* genome database [29]. The *NPF* sequences in *B. rapa* and *B. oleracea* were acquired from the *Brassica* Database (BRAD, http://brassicadb.org/brad).

### 4.2. Characterization of the NPF Family Genes

Gene length, protein length and intron number were analyzed using the Ensmbl-Plants search program (http://plants.ensembl.org/). The MW, PI, and GRAVY values were calculated using the ProtParam tool (https://web.expasy.org/protparam/). Subcellular localization was predicted by the WoLF PSORT server (https://wolfpsort.hgc.jp/). The structure of the NPF family genes were obtained based on the alignments of their genomic sequences and the coding sequences, and were visualized by TBtools software [56]. To identify potential conserved motifs in *B. napus* and *Arabidopsis*, the Multiple Expectation Maximization for Motif Elicitation program (MEME, http://meme-suite.org/tools/meme) was used with the following parameter settings: the optimum motif width was 6–50, and the maximum number of motifs was 9 [57].

### 4.3. Chromosomal Location, Collinearity Relationships and Gene Duplication Analysis

The chromosomal locations of the *BnaNPF* family genes were determined from the *B. napus* database. Gene duplication events and collinearity relationships were analyzed using Multiple Collinearity Scan toolkit (MCScanX) [58]. The results were displayed using Circos [59]. The criteria for analyzing potential gene duplications were: (a) length of alignable sequence covers >75 % of longer gene, and (b) similarity of aligned regions >75 %. These criteria were also used to screen the homologous *NPF* genes between *Arabidopsis*, *B. napus*, *B. rapa* and *B. oleracea* based on their coding sequences (CDS). The syntenic maps were constructed using MCScanX to exhibit the synteny relationship of the orthologous *NPF* genes from *Arabidopsis*, *B. napus*, *B. rapa* and *B. oleracea*. To exhibit the synteny relationship of the orthologous *NPF* genes from *Arabidopsis*, *B. napus*, *B. rapa* and *B. oleracea*, the syntenic maps were constructed using MCScanX [58].

### 4.4. Phylogenetic and Evolutionary Pressure Analysis of the BnaNPF Family Genes

Multiple sequence alignments of all predicted NPF proteins from *Arabidopsis* and *B. napus* were performed using ClustalW software. Unroot phylogenetic trees were constructed using the full protein sequences of NPF genes in *Arabidopsis* and *B. napus* by MEGA 5.1 with neighbor joining (NJ) method, and a bootstrap analysis was conducted using 1000 replicates [60]. The Ks, Ka and Ka/Ks values were calculated based on CDS alignments of the *NPF* gene coding sequences of each paralogous pair to analyze the evolutionary pressure using PAL2NAL program (http://www.bork.embl.de/pal2nal/index.cgi) [61].

### 4.5. Heatmap and Coexpression Networks of the BnaNPF Family Genes

Heatmap and coexpression networks of the *BnaNPF* genes were constructed based on the RNA-seq data. Fourteen-day-old plants were treated with nutrient solution free of N, P or K, or with nutrient solution containing 6 mM NaNO_3_ (control) and NH_4_Cl, respectively. Fully expanded leaves and roots were sampled, and total RNA was extracted and used for transcriptome sequencing. A total of 18 RNA samples were subjected to the Illumina HiSeq 2500 platform (Illumina, San Diego, CA, USA). The heatmap of *BnaNPF* family genes was drawn using TBtools with clustering and normalization [56]. The interaction relationships of each gene pair in the *BnaNPF* family were calculated based on the corresponding transcript abundance (FPKM value) under N-sufficient (6 mM N) and N-free (0 mM N) treatments using DeGNServer (http://plantgrn.noble.org/DeGNServer/) [62]. The parameter settings were (1) value-based co-expression network type, (2) Pearson correlation estimation method, and (2) association cutoff > 0.6. Gene coexpression networks were visualized by Cytoscape [63].

### 4.6. Cis-Regulatory Element and Transmembrane Domain Analysis

The 2.0-kb upstream sequences of the initiation codon of the core NPF family genes in *B. napus* were obtained from the CNS-Genoscope database [29]. These sequences were analyzed in plantCARE (http://bioinformatics.psb.ugent.be/webtools/plantcare/html/) to obtain putative CREs, and all the CREs except the TATA-box and CAAT-box were illustrated using WordArt (https://wordart.com/). The phytohormone response CREs and N status response CREs were displayed using Gene Structure Display Sever 2.0 (http://gsds.cbi.pku.edu.cn/). The transmembrane domains of the core *BnaNPF* genes were analyzed using TMHMM 2.0 (http://www.cbs.dtu.dk/services/TMHMM/).

### 4.7. Plant Materials and Growth Condition

The seedlings of a rapeseed cultivar “ZS11” were hydroponically cultured in an environmentally controlled growth room at 22 °C under a 16 h light/8 h dark cycle. The light density was 300–230 μmol proton m^−2^ s^−1^. Seeds were soaked in deionized water in the dark for 2 days and subsequently transferred to a net floating on 0.5 mM CaCl_2_ solution for 3 days. The seedlings were then grown in a nutrient solution (pH 5.8) according to our previous report [31]. The solution contained NH_4_NO_3_ 3.0 mM, NaH_2_PO_4_·2H_2_O 1.0 mM, MgSO_4_·7H_2_O 2.0 mM, KCl 2.0 mM, CaCl_2_ 3.24 mM, H_3_BO_3_ 46.0 μM, MnCl_2_·4H_2_O 9.14 μM, Na_2_MoO_4_·2H_2_O 0.5 μM, ZnSO_4_·7H_2_O 0.77 μM, CuSO_4_·5H_2_O 0.32 μM and EDTA-iron (Fe) 25.0 μM. The nutrient solution was refreshed every 3 days. For nutrient deficiency treatments, half of the 14-day-old seedlings were then transferred to a solution free of N, P or K for seven days, while the other half remained in normal nutrient supply as a control. For the ammonium (NH_4_^+^) toxicity treatment, 14-day-old plants were treated with nutrient solution containing 6 mM NaNO_3_ (control) and NH_4_Cl for 9 days, respectively. Shoots and roots of the seedlings from three nutrient deficiency treatments and NH_4_^+^ toxicity treatment were harvested for RNA-seq analysis. Roots, hypocotyl, basal node, petioles, fully expanded leaves and newly-grown leaves of the seedling from N deprivation treatment were individually harvested for qRT-PCR analysis. All the samples were frozen immediately in liquid N and then stored at −80 °C. Each sample included three independent biological replicates.

### 4.8. Quantitative Real-Time PCR Analysis

An RNeasy Plant Mini Kit (Qiagen, Promega, Shanghai, China) was used to isolate total RNA from various tissues, and cDNA was synthesized using First Strand cDNA synthesis kit (Toyobo, Osaka, Japan). The qRT-PCR was carried out in a 10 μL volume containing 2 μL cDNA, 0.2 μL forward primer, 0.2 μL reverse primer, 5 μL Hieff qPCR SYBR Green Master Mix (Low Rox) and 2.6 μL ddH_2_O. The thermal cycle was as follow: Stage 1: 95 °C for 5 min; Stage 2: 40 cycles of 95 °C for 10 s, 60 °C for 20 s and 72 °C for 20 s; Stage 3: 95 °C for 15 s; Stage 4: 60 °C for 1 min; Stage 5: 95 °C for 10 s. The qRT-PCR were carried out on the QuantStudio 6 Flex instrument (Life Technologies, Carlsbad, CA, USA) with the gene-specific primers listed in Table S1. Relative expression values of the target genes were calculated according to the 2*^−ΔΔCt^* method with a housekeeping gene *EF1-α* (Accession number: DQ312264) as an internal standard.

### 4.9. Sub-Cellular Localization Analysis

For subcellular localization, the coding regions of the four *BnaNPF* genes without stop codons amplified from “ZH11” were ligated separately into the transient expression vector (PM999) driven by the CaMV35S promoter [63]. All *35S*::*BnaNPF-GFP* fusion construct and cell membrane localization *35S*::*AtNIP5;1*-*mCherry* was co-expressed into protoplasts of *Arabidopsis* [64]. The fluorescence signals of BnaNFP-GFP and AtNIP5;1-mCherry protein in *Arabidopsis* protoplasts were checked using Leica SP8 lazer confocal microscopy system (Leica Microsystems, Shanghai, China) after transformation for 12~16 h.

### 4.10. Statistical Analysis of Data

For statistical analyses, significant differences were determined by T-test using SPSS 22 (IBM, Chicago, IL, USA) at * *P* < 0.05 and ** *P* < 0.01.

## 5. Conclusions

In summary, we identified 95, 93 and 193 *NPF* family genes on the whole genome of *B. rapa*, *B. oleracea* and *B. napus*, respectively. Our analysis of *BnaNP*F sequences together with the available functional studies suggest that NPFs are versatile transporters in *B. napus*, and may be involved in regulating tolerance of *B. napus* to multiple nutrient stresses. Our results give the first step in the complex genetic dissection of the *BnaNPF* family, and provide the fundamental information for further research of the specific functions of a single gene in this gene family in *B. napus*.

## Figures and Tables

**Table 1:** Copy number of the NRT1/PTR family (NPF) in *Arabidopsis* and three *Brassica* species.

Item	*Arabidopsis thaliana*	*Brassica rapa*	*Brassica oleracea*	*Brassica napus*
NPF1	3	4	6	10
NPF2	14	22	21	42
NPF3	1	3	3	6
NPF4	7	8	8	19
NPF5	16	36	32	73
NPF6	4	7	7	13
NPF7	3	6	6	10
NPF8	5	9	10	20
Total	53	95	93	193

## Supplementary Materials

The following are available online at https://www.mdpi.com/1422-0067/21/17/5947/s1, Figure S1: Gene structure of the *NPF* family in *Arabidopsis* and *Brassica napus*. Green boxes indicate UTR regions, yellow boxes indicate exons, and black lines indicate introns. **Figure S2**: Conserved motifs of the NPF family protein in *Arabidopsis* and *Brassica napus*. (**A**) Motif characterization of BnaNPF proteins. The motifs, numbers 1-9, are displayed in different colored boxes. (**B**) the sequence information for each motif. **Figure S3**: Chromosomal locations of *NPF* family in *Brassica napus*. The 193 *BnaNPF* genes were mapped to the 19 chromosomes of *B. napus* based on the exact chromosomal information available in the database. The red fonts indicate the position of the corresponding *BnaNPF* genes on each chromosome. The diagram was drawn using the Mapchart software. Bar = 10 Mb. **Figure S4**: Transmembrane domain analysis of the 10 core *NPF* genes in *Brassica napus*. The diagram was drawn based the full-length of the core BnaNPF protein sequences using the TMHMM program. The red regions or boxes indicate the transmembrane domains. **Table S1**: Primers used for quantitative real-time PCR used in this study. **Table S2**: Characteristics of the *NPF* family members in *Brassica napus* and subcellular localization prediction. **Table S3**: Non-synonymous (Ka) and synonymous (Ks) nucleotide substitution rates for *NPF* coding loci in *Arabidopsis* and *Brassica napus*. **Table S4**: FPKM of the *NPF* family in leaves and roots of *Brassica napus* under nitrogen, phosphorus, potassium stresses and ammonium toxicity.

## Abbreviations

N	Nitrogen
P	Phosphorus
K	Potassium
NO_3_^−^	Nitrate
NH_4_^+^	Ammonium
NPF	NITRATE TRANSPORTER 1 (NRT1)/PEPTIDE TRANSPORTER (PTR) family (NPF)
DEG	Differentially expressed gene
CRE	*Cis*-regulatory element
TMD	Transmembrane domain
TF	Transcription factor
FPKM	Fragments per kilobase of transcript per million fragments mapped
