# Supplementary material for: Genome-Wide Systematic Characterization of the NPF Family Genes and Their Transcriptional Responses to Multiple Nutrient Stresses in Allotetraploid Rapeseed
Source: Int J Mol Sci. 2020 Aug 19;21(17):5947. doi: 10.3390/ijms21175947 (PMC7504168; doi:10.3390/ijms21175947)
Supplement: Supplementary file 1 [file ijms-21-05947-s001.zip › Table S1.docx]

**Table S1. Primers used for quantitative real-time PCR in this study**

| **Gene name** | **Forward primer** | **Reverse primer** |
| --- | --- | --- |
| *BnaC03.NPF2;7* | AACCCCTGTGACACTGCTT | AGACAATGGCGGTTACACTT |
| *BnaC08.NPF2;9a* | AGACGTCTCGGTTCAGGAAG | AAACAAGCCTGCTCCAACTC |
| *BnaA07.NPF2;13a* | ACAAAGCGGCGGTGATAAAA | CCCATGTGTCGGTCCATTTT |
| *BnaC06.NPF2;13a* | TCCGGATCACATGAGAAGCA | ACACCCAAAATCGCGATCAG |
| *BnaA07.NPF4;6* | GCTTTAGGAGTGGGAGGGAT | TCCCCATTCCCATCCTTTGT |
| *BnaC06.NPF4;6* | GTCATTACCGTCGCATGGAG | TCCCCATTCCCATCCTTTGT |
| *BnaA05.NPF7;3* | CCCATTTGGCTCTGCACTATC | GCTCGAGAACTCTGCGGTAT |
| *BnaC05.NPF7;3a* | GGTTCGCTCTTCTCCAACAC | TGCCTCCACCGATGATTTCT |
| *BnaC05.NPF7;3b* | CTCATTGGTCGGAGCGTTTC | GTTCCCCAATGCGATCAAGT |
| *EF1-α* | GCCTGGTATGGTTGTGACCT | GAAGTTAGCAGCACCCTTGG |
