# Supplementary figures and images for: Genome-Wide Systematic Characterization of the NPF Family Genes and Their Transcriptional Responses to Multiple Nutrient Stresses in Allotetraploid Rapeseed
Source: Int J Mol Sci. 2020 Aug 19;21(17):5947. doi: 10.3390/ijms21175947 (PMC7504168; doi:10.3390/ijms21175947)

## Figure S1

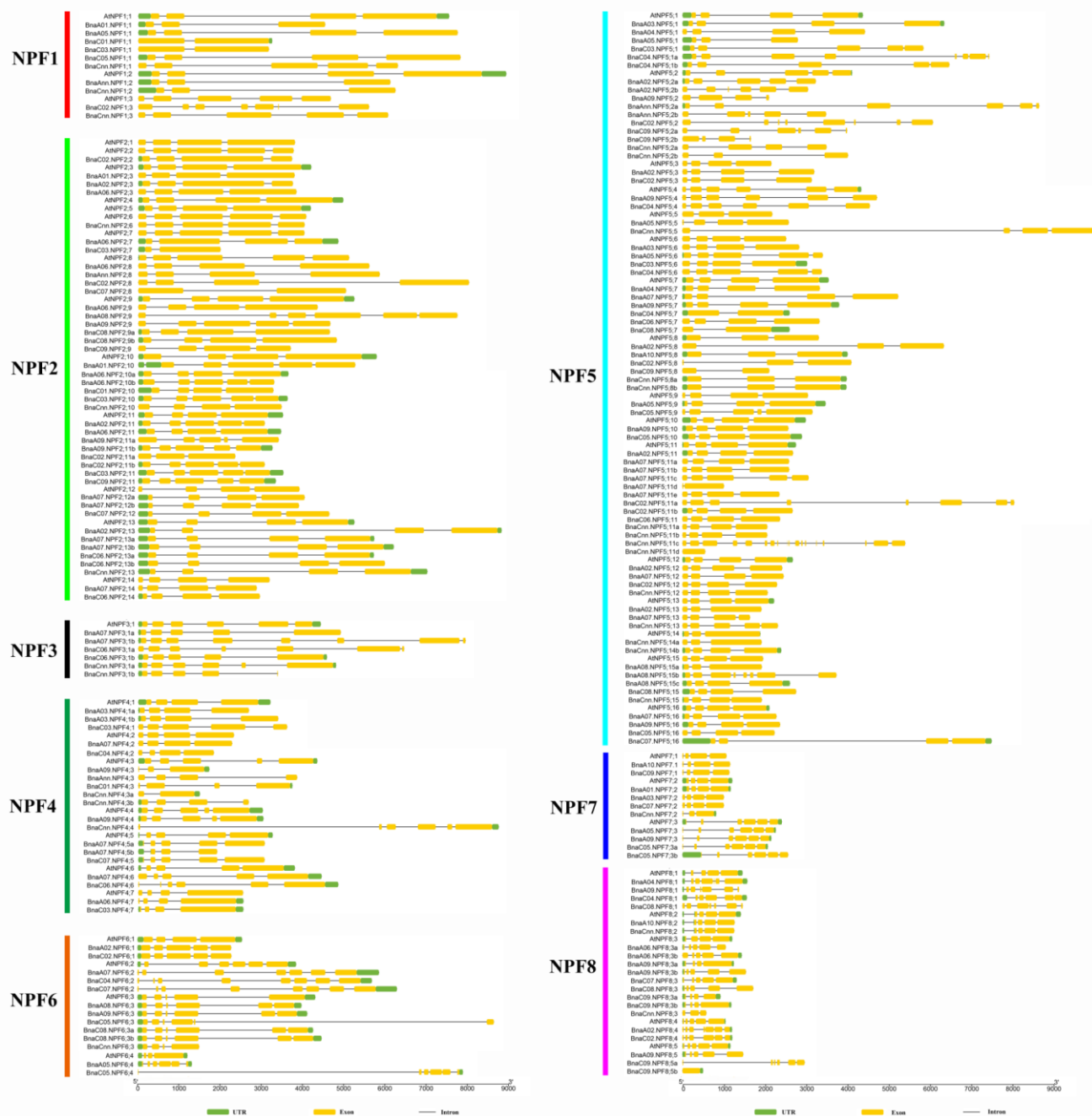

Figure S2

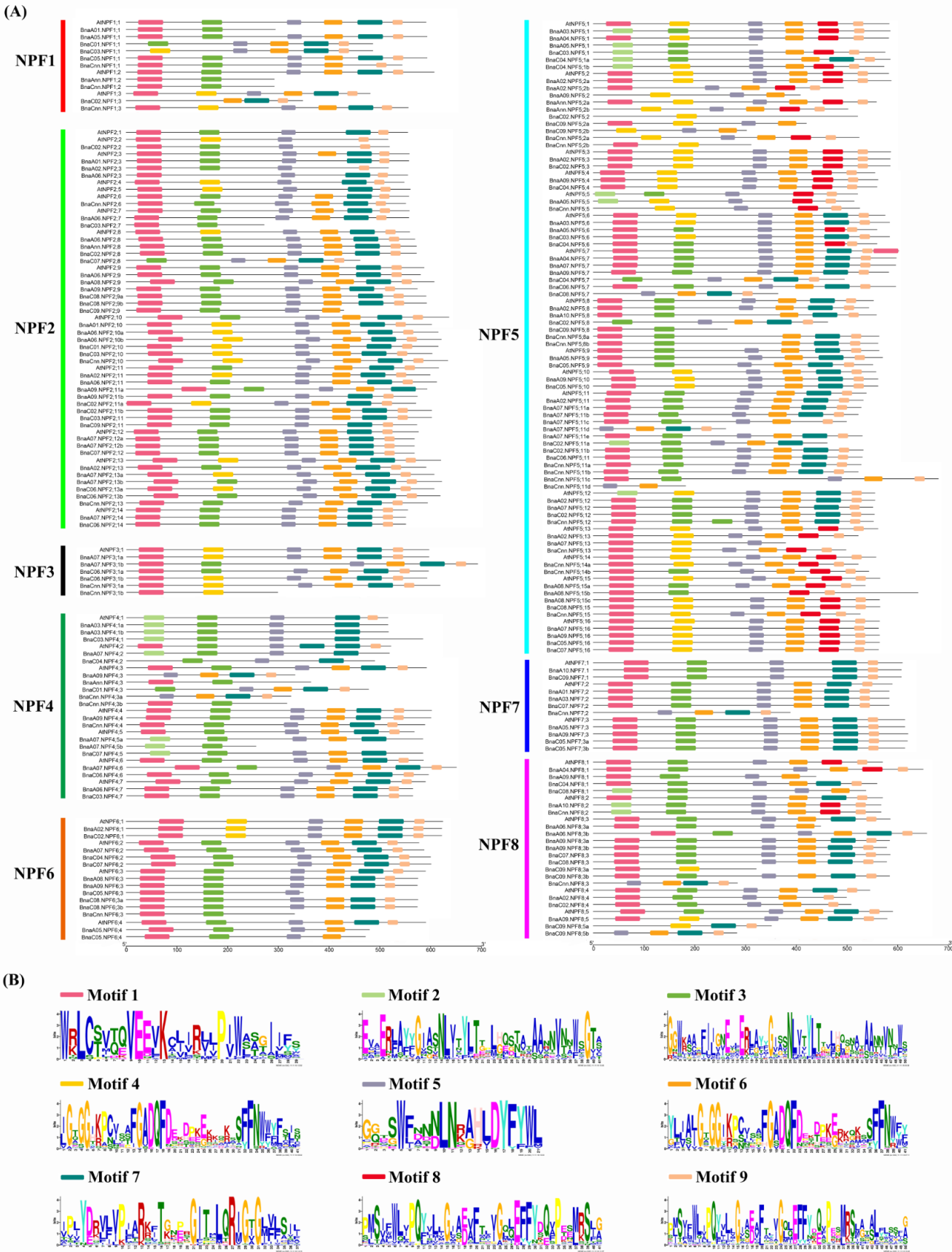

Figure S3

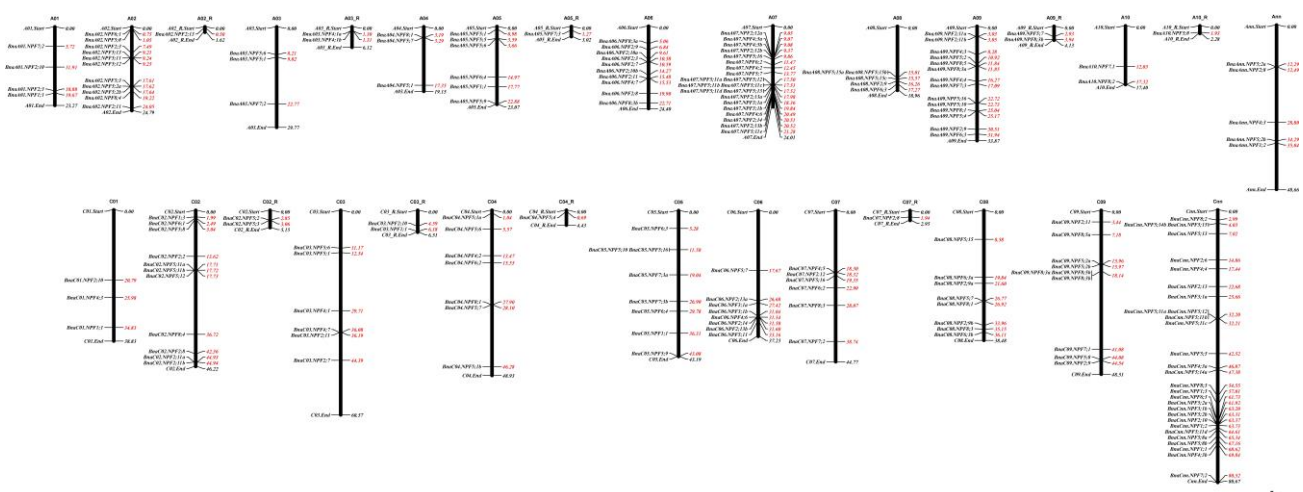

Figure S4

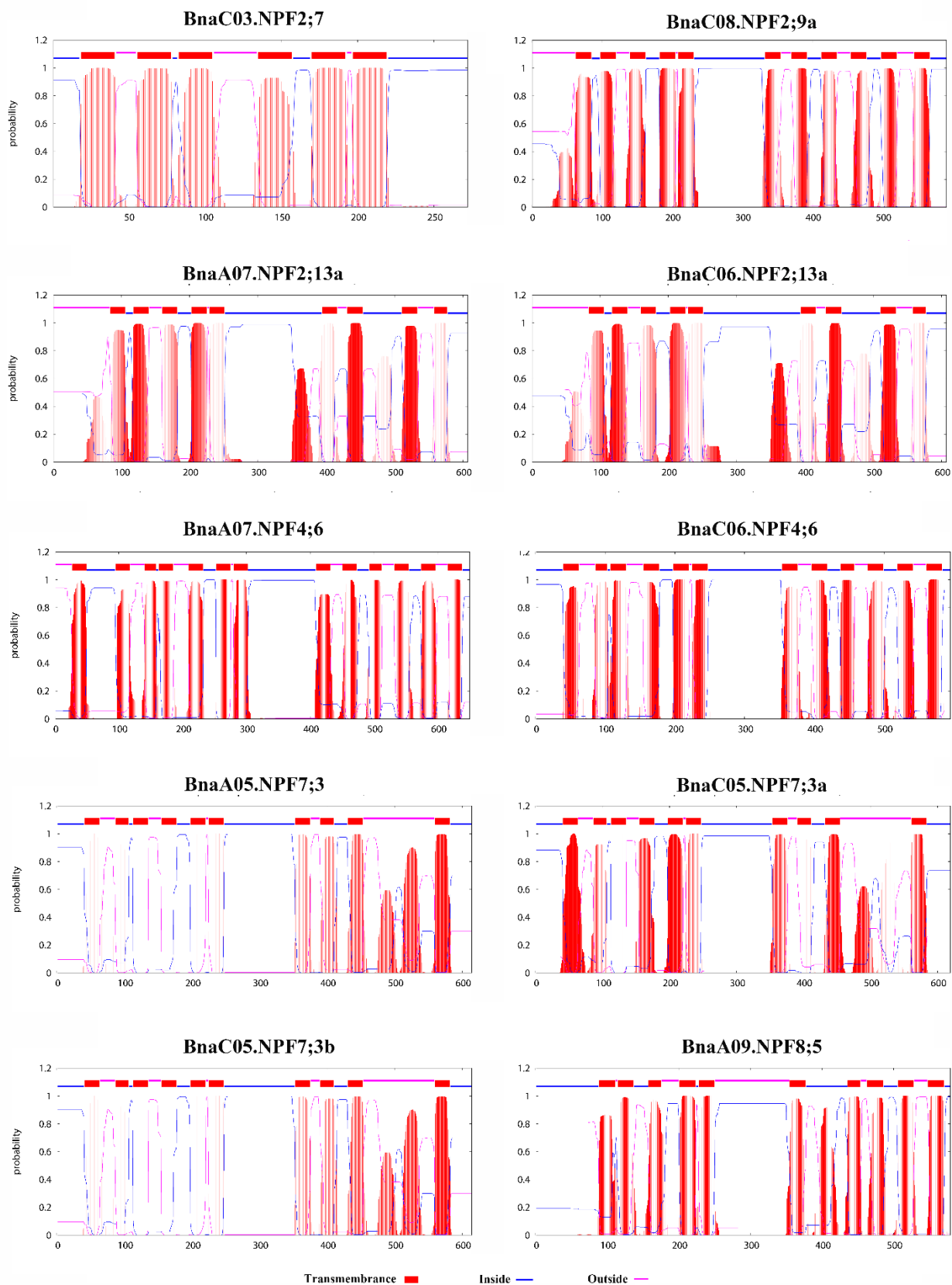

Supplement: Supplementary file 1 [file ijms-21-05947-s001.zip › Figures S1-S4.pdf]
